# Risks of Sulpiride-Induced Parkinsonism in Peptic Ulcer and Gastroesophageal Reflux Disease Patients in Taiwan: A Nationwide Population-Based Study

**DOI:** 10.3389/fphar.2020.00433

**Published:** 2020-04-24

**Authors:** Cheng-Yu Wei, I-Shiang Tzeng, Mei-Chen Lin, Yung-Hsiang Yeh, Chung Y. Hsu, Woon-Man Kung

**Affiliations:** ^1^ Department of Neurology, Chang Bing Show Chwan Memorial Hospital, Changhua County, Taiwan; ^2^ Department of Exercise and Health Promotion, College of Education, Chinese Culture University, Taipei, Taiwan; ^3^ Management Office for Health Data, China Medical University Hospital, Taichung, Taiwan; ^4^ College of Medicine, China Medical University, Taichung, Taiwan; ^5^ Digestive Disease Center, Chang Bing Show Chwan Memorial Hospital, Changhua County, Taiwan; ^6^ Graduate Institute of Clinical Medical Science, College of Medicine, China Medical University, Taichung, Taiwan

**Keywords:** sulpiride, drug-induced parkinsonism, peptic ulcer disease, gastroesophageal reflux disease, population-based study

## Abstract

**Background:**

Sulpiride is a highly selective dopamine D2 receptor antagonist and is commonly used in psychiatric disorders, Tourette syndrome, peptic ulcer disease (PUD), and gastroesophageal reflux disease (GERD). However, sulpiride has been recognized as a potential cause of drug-induced parkinsonism (DIP) for a long time. In this study, we aimed to focus on analysis of sulpiride-induced parkinsonism (SIP) in PUD and GERD patients based on a nationwide population.

**Methods:**

Data were obtained from the Taiwan’s National Health Insurance Research Database. The study enrolled 5,275 PUD or GERD patients, of whom were divided into two groups, based on their exposure (1,055 cases) or non-exposure (4,220 cases) to sulpiride.

**Results:**

During the study period (2000–2012), the incidence rate of parkinsonism was 261.5 and 762.2 per 100,000 person-years in the control and sulpiride-treated groups, respectively. For patients with at least 14 days of prescription for sulpiride, the adjusted hazard ratio (aHR) was 2.89, 95% confidence interval (CI): 2.04-4.11. Patients with age more than 65 years (aHR = 4.99, 95% CI = 2.58-9.65), hypertension (aHR = 2.39, 95% CI = 1.49-3.82), depression (aHR = 2.00, 95% CI = 1.38-2.91), and anxiety (aHR = 1.45, 95% CI = 1.01-2.09) had significant higher risk of developing parkinsonism. An average annual cumulative sulpiride dose > 1,103 mg was accompanied by the greatest risk of SIP; sulpiride use for ≥ 9 days is a cut-off point for predicting future SIP.

**Conclusion:**

At the population level, sulpiride may be frequently prescribed and apparently effective for PUD and GERD. SIP is associated with older age, hypertension, depression or anxiety comorbidities. Physicians should be aware of the neurogenic adverse effects, even when the drug is only used in low-dose or a short duration.

## Introduction

Sulpiride is a substituted benzamide and is classified as a low potent atypical antipsychotics. It is a weak but highly selective dopamine D2 receptor antagonist (Jenner et al., 1982; Caley and Weber, 1995; Mauri et al., 1996). It is used to treat a variety of psychiatric disorders including depression, somatoform disorders, and schizophrenia (Kato, 1993; Mucci et al., 1995; Mauri et al., 1996; Rouillon et al., 2001). Sulpiride is one of the neuroleptics in treating tics for Tourette syndrome (Eddy et al., 2011). In the field of gastroenterology, it is also used as an antiemetic and antidyspeptic drug for peptic ulcer disease (PUD) and gastroesophageal reflux disease (GERD) for more than 50 years (Caldara et al., 1978; Lam et al., 1979; Tatsuta et al., 1986; Trabucchi et al., 1991; Desai and Parmar, 1994).

Sulpiride is commonly used in Asia, Europe, Central America, South America, and South Africa. However, it is not approved in the United States, Canada, or Australia (Caley and Weber, 1995). The safety profile of sulpiride is similar to other typical antipsychotics. Its common adverse effects (1 and <10% by the Council for International Organizations of Medical Sciences (CIOMS) frequency rating) include sedation, drowsiness, insomnia, weight gain, increased hepatic enzyme, constipation, maculo-papular rash, hyperprolactinemia, breast pain, galactorrhoea, and extrapyramidal disorder (Standish-Barry et al., 1983; Gerlach et al., 1985; Lepola et al., 1989; Mauri et al., 1996). The extrapyramidal manifestations caused by sulpiride include dystonia, akathisia, parkinsonism, and tremor (Eapen et al., 1993; Mauri et al., 1996; Lai et al., 2014). Recently, two big data-based studies and one meta-analysis have focused on drug-induced parkinsonism (DIP) (Martino et al., 2018; Byun et al., 2019; Kim et al., 2019). The first population-based study concluded that use of propulsives and antipsychotics including sulpiride had a significant association with the increased risk of DIP, depending on recency and cumulative dose (Kim et al., 2019). Another population-based research found that annual prevalence of DIP has increased, and the usage of specific offending medications is the major cause (Byun et al., 2019). In the meta-analysis study focused on second-generation antipsychotics, the prevalence estimates are of 15.3% for acute dystonia, 16.4% for akathisia, 29.3% for parkinsonism, and 28.2% for tremor induced by sulpiride (Martino et al., 2018).

PUD and GERD are popular gastrointestinal disorders that can cause troublesome symptoms, and have a significant impact on quality of life (Lanas and Chan, 2017; Yamasaki et al., 2018). However, to the best of our knowledge, no population-based analyses have been performed for sulpiride-induced parkinsonism (SIP) in these subjects. This study aimed to investigate the risk factors and the cumulative daily dose associated with SIP.

## Methods

### Data Source

Taiwan built a single-payer National Health Insurance program (Taiwan NHI) in 1995, and nearly 99% of Taiwan’s citizens were enrolled in the program currently. The database named National Health Insurance Research Database (NHIRD), which included the detailed records of outpatients, hospitalization, treatment, prescription, and other medical services for each patient. In this study, we conducted the analyses by using Longitudinal Health Insurance Database (LHID), which is the subset database and randomly selected 1 million study subjects from NHIRD. The privacy of each patient was protected by encrypting the identification number before the database is released. All diagnoses in Taiwan NHI are coded according to the International Classification of Disease, Ninth Revision, Clinical Modification (ICD-9-CM). The Research Ethics Committee of China Medical University and Hospital in Taiwan approved the study (CMUH-104-REC2-115-R3).

### Study Population

To clarify the association between PUD or GERD patients with or without sulpiride and parkinsonism, we defined two cohorts: PUD or GERD patients (ICD-9-CM 533, 530.11, 530.81) with at least 14 days of prescription for sulpiride (ATC code: N05AL01) (case), and PUD or GERD patients without any sulpiride usage record (control). The index date was defined as the starting date of receiving sulpiride therapy, and followed up until patients firstly diagnosed with Parkinson’s disease (PD, ICD-9-CM 332) or parkinsonism (ICD-9-CM 333, excluding 333.1-333.8), or withdrawn from NHIRD, or after the date December 31, 2013.

**Figure 1:**
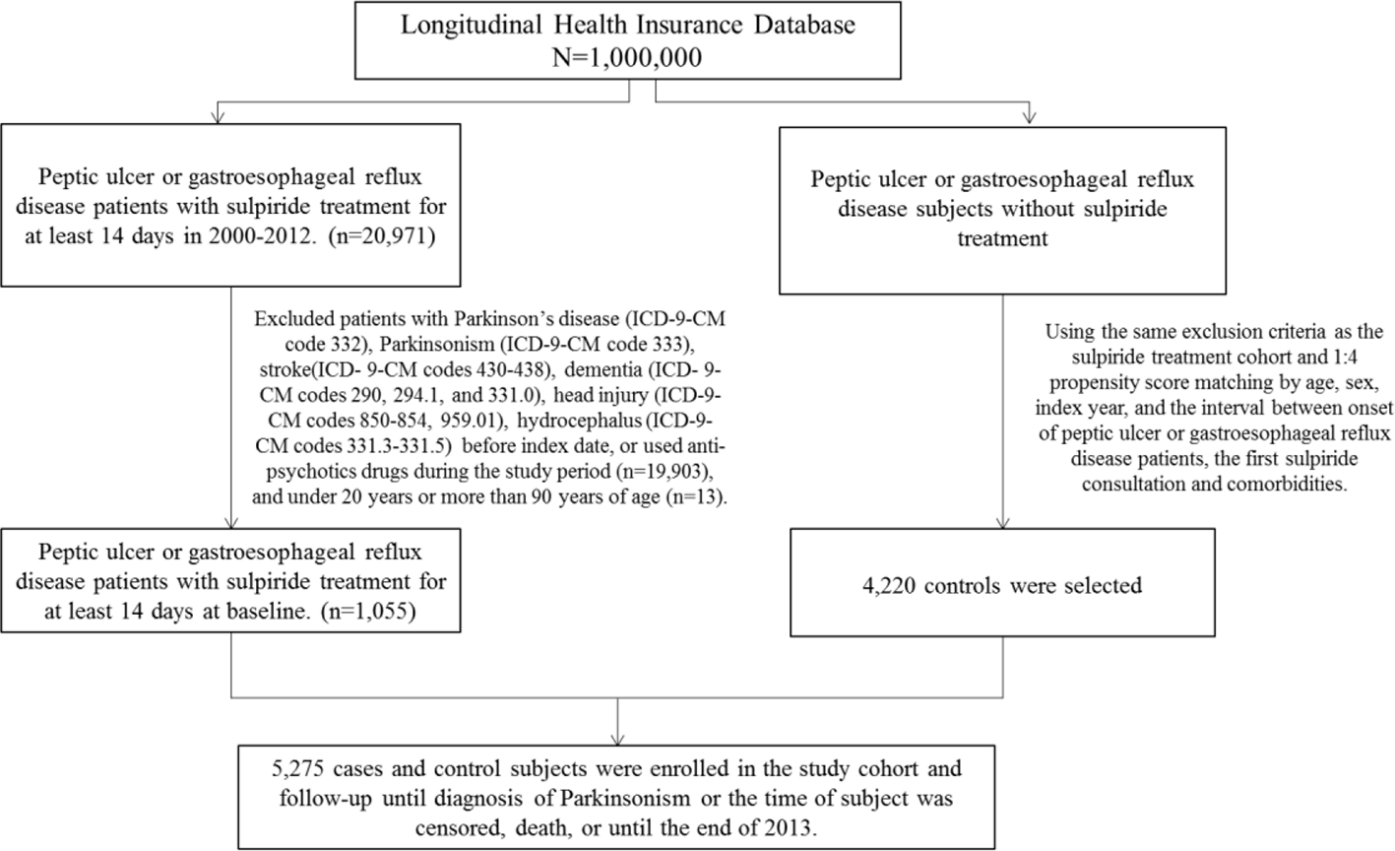
Selection process of patients in the two study cohorts.

The comorbidities were important confounding factors in NHIRD studies. We defined comorbidities with at least twice outpatients or once hospitalization of diagnoses before index date, including hypertension (ICD-9-CM 401-405), diabetes (ICD-9-CM 250), hyperlipidemia (ICD-9-CM 272), depression (ICD-9-CM 296.2, 296.3, 300.4, 311), anxiety (ICD-9-CM 300.00), and sleep disorder (ICD-9-CM 307.4, 780.5). Patients with PD (ICD-9-CM 332), parkinsonism (ICD-9-CM 333), stroke (ICD-9-CM 430-438), dementia (ICD-9-CM 290, 294, 331.0), head injury (ICD-9-CM 310.2, 800, 801, 803, 804, 850-854, and 959.01), or hydrocephalus (ICD-9-CM 331.3, 331.4, 331.5, 741.0, 742.3) before the index date, those who used antipsychotics (ATC code N05A) during the study period, and patients with age < 20 years or > 90 years were excluded in our study. Each case was propensity matched by age, gender, index year, first sulpiride prescription date, hypertension, diabetes, hyperlipidemia, depression, anxiety, and sleep disorder with four controls (**Figure 1**).

### Statistical Analysis

To compare the difference between sulpiride and the comparison cohorts, we use two-sample t-test for continuous variable and chi-square test for categorical variable. The incidence rate (per 100,000 person years) of parkinsonism was calculated for both cohorts. The Kaplan-Meier method was used to plot the cumulative incidence curves for each cohort, and log rank test was applied to assess the difference of two survival curves. We estimated hazard ratios (HRs), adjusted hazard ratio (aHR), and 95% confidence intervals (CIs) for risk of parkinsonism in sulpiride, and the comparison cohort by using crude and adjusted Cox proportional hazard models. We also stratified the annual mean sulpiride prescription days, annual mean sulpiride dosage, cumulative defined daily dose (cDDD) of sulpiride into two levels by median, and calculated the risk of parkinsonism in each group.

All statistical analyses were performed using SAS statistical software, version 9.4 (SAS Institute Inc., Cary, NC). The figure of cumulative incidence curve was plotted by R software. The significant level was set at less than 0.05 for two-side testing of p-value.

## Results

We totally enrolled 5,275 study subjects (**Table 1**), including 1,055 cases and 4,220 controls in this study. Among the patients, about 68.7% were female and the dominant age group was 45 to 65 years. The mean ages were 52.9 and 52.3 years in control and case group, respectively. The distribution of demographic and comorbidities had no significant difference between two groups after propensity score matching (p > 0.05).

**Table 1:** Demographic characteristics, comorbidities of PUD or GERD patients with or without sulpiride in Taiwan during 2000-2012.

Variable	PUD or GERD patients			p-value
	Total	Non-Sulpiride	Sulpiride	
	N=5275	n=4220	n=1055	
	n	n (%)/mean ± SD	n (%)/mean ± SD	
**Gender**				0.767
Female	3620	2900 (68.7)	720 (68.2)	
Male	1655	1320 (31.3)	335 (31.8)	
**Age at baseline**				0.212
<45	1607	1263 (29.9)	344 (32.6)	
45-65	2505	2025 (48)	480 (45.5)	
>65	1163	932 (22.1)	231 (21.9)	
Mean(SD) ‡		52.9 (14.5)	52.3 (14.8)	0.274
**Baseline comorbidity**				
Hypertension	2315	1857 (44.0)	458 (43.4)	0.729
Diabetes	1334	1072 (25.4)	262 (24.8)	0.704
Hyperlipidemia	2129	1710 (40.5)	419 (39.7)	0.633
Depression	1530	1205 (28.6)	325 (30.8)	0.150
Anxiety	2132	1695 (40.2)	437 (41.4)	0.457
Sleep disorder	3121	2518 (59.7)	603 (57.2)	0.138

Chi-square test, Student’s t-test‡.

SD, standard deviation.

**Table 2** presented the risk factors of parkinsonism among PUD or GERD patients. Patients with at least 14 days of prescription for sulpiride (aHR = 2.89, 95% CI = 2.04-4.11), age more than 65 years (aHR = 4.99, 95% CI = 2.58-9.65), hypertension (aHR = 2.39, 95% CI = 1.49-3.82), depression (aHR = 2.00, 95% CI = 1.38-2.91), and anxiety (aHR = 1.45, 95% CI = 1.01-2.09) had significant higher risk of developing parkinsonism after adjusted by age, gender, and comorbidities.

**Table 2:** Cox model measured hazard ratio and 95% confidence intervals of parkinsonism associated with or without sulpiride and covariates among PUD or GERD patients.

Characteristics	Event no.	Crude		Adjusted	
	(n=131)	HR (95% CI)	p-value	HR (95% CI)	p-value
**Sulpiride**					
No	78	Ref.		Ref.	
Yes	53	2.91 (2.06-4.13)	<0.001	2.89 (2.04-4.11)	<0.001
**Gender**					
Female	85	Ref.		Ref.	
Male	46	1.18 (0.82-1.68)	0.374	1.13 (0.78-1.62)	0.515
**Age at baseline**					
<45	13	Ref.		Ref.	
45-65	48	2.64 (1.43-4.87)	0.002	1.72 (0.90-3.29)	0.099
>65	70	9.39 (5.19-16.99)	<0.001	4.99 (2.58-9.65)	<0.001
**Baseline comorbidity**					
Hypertension	102	5.14 (3.40-7.78)	<0.001	2.39 (1.49-3.82)	<0.001
Diabetes	49	2.03 (1.42-2.89)	<0.001	1.10 (0.75-1.61)	0.637
Hyperlipidemia	71	2.02 (1.43-2.85)	<0.001	1.04 (0.72-1.51)	0.842
Depression	53	1.97 (1.39-2.79)	<0.001	2.00 (1.38-2.91)	<0.001
Anxiety	61	1.68 (1.19-2.38)	0.0032	1.45 (1.01-2.09)	0.044
Sleep disorder	76	1.29 (0.91-1.83)	0.1601	0.78 (0.53-1.14)	0.205

HR, hazard ratio; CI, confidence interval;

Adjusted HR: adjusted for age, gender, and comorbidities in Cox proportional hazards regression.

**Figure 2:**
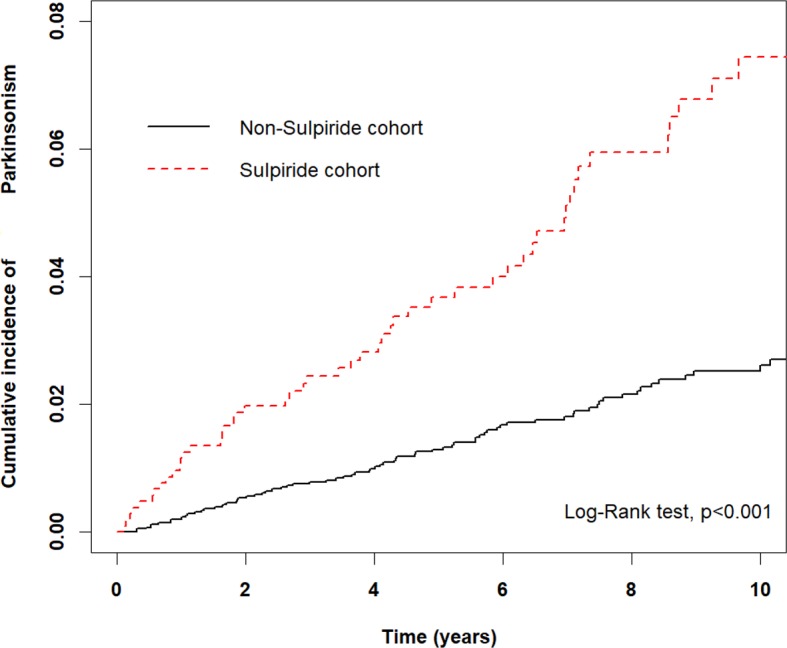
Kaplan-Meir method to determine the cumulative incidence of parkinsonism compared between the sulpiride and non-sulpiride cohort.

In our study, the incidence rate of DIP in PUD or GERD patients under sulpiride exposure was 762.2 per 100,000 person-years. **Figure 2** demonstrated significant higher cumulative incidence of parkinsonism in the sulpiride cohort, compared to the non-sulpiride cohort (p < 0.001).

The multivariate stratified analysis was conducted and shown in **Table 3**. The incidence rate of parkinsonism was 261.5 and 762.2 per 100,000 person-years in the control and sulpiride-treated group respectively. The sulpiride treatment among PUD or GERD patients increased the risk of parkinsonism; female (aHR = 3.12, 95% CI = 2.02-4.81), male (aHR = 2.53, 95% CI = 1.39-4.60), age less than 45 years (aHR = 8.79, 95% CI = 2.69-28.73), age 45 to 65 years (aHR = 3.85, 95% CI = 2.17-6.84), age more than 65 years (aHR = 1.72, 95% CI = 1.02-2.90), those with hypertension (aHR = 2.24, 95% CI = 1.49-3.39), diabetes (aHR = 2.33, 95% CI = 1.26-4.30), hyperlipidemia (aHR = 2.03, 95% CI = 1.22-3.38), depression (aHR = 2.26, 95% CI = 1.29-3.97), anxiety (aHR = 1.87, 95% CI = 1.07-3.25), and sleep disorder (aHR = 2.41, 95% CI = 1.50-3.87).

**Table 3:** Incidence rates, hazard ratio and confidence intervals of parkinsonism in different stratification.

Variables	Control			Case			Case VS. Control			p-value
	N = 4220			N = 1055			Crude HR	p-value	Adjusted HR	
	Event	Person years	IR	Event	Person years	IR	(95% CI)		(95% CI)	
**Overall**	78	29828	261.5	53	6954	762.2	2.91 (2.06-4.13)	< 0.001	2.89 (2.04-4.11)	< 0.001
**Gender**										
Female	50	20454	244.5	35	4714	742.4	3.04 (1.97-4.68)	< 0.001	3.12 (2.02-4.81)	< 0.001
Male	28	9374	298.7	18	2239	803.9	2.69 (1.49-4.87)	0.001	2.53 (1.39-4.60)	0.002
**Age at baseline**										
<45	4	9872	40.5	9	2476	363.5	9.11 (2.80-29.60)	< 0.001	8.79 (2.69-28.73)	< 0.001
45-65	24	14120	170	24	3185	753.5	4.39 (2.49-7.74)	< 0.001	3.85 (2.17-6.84)	< 0.001
>65	50	5836	856.7	20	1293	1547.3	1.81 (1.08-3.04)	0.025	1.72 (1.02-2.90)	0.042
**Baseline comorbidity**										
Hypertension	67	12154	551.3	35	2807	1246.8	2.26 (1.50-3.40)	< 0.001	2.24 (1.49-3.39)	< 0.001
Diabetes	34	6841	497	15	1499	1001.0	1.98 (1.08-3.64)	0.028	2.33 (1.26-4.30)	0.007
Hyperlipidemia	49	11012	445	22	2549	862.9	1.94 (1.17-3.20)	0.010	2.03 (1.22-3.38)	0.006
Depression	33	7386	446.8	20	2029	985.7	2.21 (1.27-3.86)	0.005	2.26 (1.29-3.97)	0.004
Anxiety	43	9936	432.8	18	2590	695.1	1.64 (0.95-2.85)	0.077	1.87 (1.07-3.25)	0.027
Sleep disorder	49	15436	317.4	27	3533	764.1	2.42 (1.51-3.87)	< 0.001	2.41 (1.50-3.87)	< 0.001

IR, incidence rates, per 100,000 person-years; HR, hazard ratio; CI, confidence interval.

Adjusted HR: adjusted for age, gender, and comorbidities in Cox proportional hazards regression.

The analyses of sulpiride usage were stratified by medication duration (per year), dosage (per year), and cumulative defined daily dose during the study period and classified by median in each group respectively (**Table 4**). Compared to patients without sulpiride, patients with sulpiride have more than 9 days per year (aHR = 4.28, 95% CI = 2.88-6.36), more than 1,103 mg per year (aHR = 4.63, 95% CI = 3.12-6.86), less than 6.75 cDDD (aHR = 2.50, 95% CI = 1.51-4.13), and more than 6.75 cDDD (aHR = 3.20, 95% CI = 2.13-4.81) during the study period with a significant higher risk of developing parkinsonism.

**Table 4:** Incidence and adjusted hazard ratio of parkinsonism stratified by duration (per year), dosage (per year), and cumulative defined daily dose of sulpiride therapy in PUD or GERD patients.

Medication exposed	Event	Person year	IR	Adjusted HR (95% CI)	p-value
Non-Sulpiride	78	29828	261.5	Ref.	
Sulpiride					
<9 days	15	3988	376.1	1.58 (0.91-2.75)	0.105
≥9 days	38	2904	1274.1	4.28 (2.88-6.36)	<0.001
<1103 mg	15	3997	375.3	1.50 (0.86-2.61)	0.151
≥1103 mg	38	2943	1291.1	4.63 (3.12-6.86)	<0.001
<6.75 cDDD	19	3070	618.8	2.50 (1.51-4.13)	<0.001
≥6.75 cDDD	34	3870	878.6	3.20 (2.13-4.81)	<0.001

IR, incidence rates, per 100,000 person-years; HR, hazard ratio; CI, confidence interval.

Adjusted HR: adjusted for age, gender, and comorbidities in Cox proportional hazards regression.

## Discussion

Sulpiride, with molecular formula C_15_H_23_N_3_O_4_S, is a selective dopamine D2 receptor antagonist. Because of low lipophilic solubility, it crosses the blood-brain barrier poorly and is mainly excreted unchanged in the urine. Excessive drug accumulation could occur in the elderly or patients with renal dysfunction (Caley and Weber, 1995; Mauri et al., 1996). As other antipsychotics, the main mechanism of SIP is to cause D2 receptor blockade in the striatum, which eventually leads to disinhibition of GABA- and encephalin-containing striatal neurons at the origin of the indirect pathway without alteration of the direct pathway, followed by disinhibition of the subthalamic nucleus (Shin and Chung, 2012). At the same time, sulpiride has higher 5-HT2A antagonism, with both of the pharmacological characteristics contributing to the risk of developing SIP. Among the second-generation antipsychotics, sulpiride has the highest prevalence of parkinsonism and tremor, even higher than that seen with haloperidol (Martino et al., 2018).

This was the first population-based study, which examined a complete picture of risk of SIP in PUD or GERD patients by using matched cohorts and a long-term follow-up period. The incidence rates of DIP in general population were 3.3 per 100,000 person-years in the United States, and 13.9 per 100,000 person-years in Korea (Savica et al., 2017; Han et al., 2019). The incidence rate of DIP in PUD or GERD patients under sulpiride exposure in Taiwan was reported in our results section, and comparable to that of United States and Korea. Therefore, physicians should be aware for the early signs of parkinsonism in the PUD or GERD patients treated with sulpiride.

In our study, subjects in all three age levels revealed significant risks of SIP (age less than 45 years, aHR = 8.79, 95% CI = 2.69-28.73; age 45 to 65 years, aHR = 3.85, 95% CI = 2.17-6.84; age more than 65 years, aHR = 1.72, 95% CI = 1.02-2.90); the elder subgroup had the highest risk (aHR =4.99, 95% CI: 2.58–9.65). Age is the most obvious risk factor for DIP because nigral dopaminergic neuronal cells degenerate with age (Shin and Chung, 2012). Female gender is considered to be a risk factor for DIP because estrogen can suppress the expression of dopamine receptors (Bedard et al., 1977; Shin and Chung, 2012). However, in our study, both male and female patients with sulpiride treatment showed a higher tendency to develop parkinsonism when compared to the control subjects.

Based on previous studies, psychological stress had been treated as a risk factor for PUD or GERD patients (Levenstein et al., 2015). Chronic stress may lead to an ulcerogenic effect on corticosterone (Zhang et al., 2012). PUD and GERD was more common among people with anxiety and mood disorders (Lim et al., 2014; Choi et al., 2018). Although sulpiride is reported to be effective to depressive or anxious symptoms (Kato, 1993), this study showed patients with depression or anxiety comorbidity had significant higher risk for developing DIP. In fact, sulpiride is not included in the evidence-based clinical practice guidelines for PUD and GERD at present (Iwakiri et al., 2016; Satoh et al., 2016). For the pharmacologic treatment of depression or anxiety comorbidity, selective serotonin reuptake inhibitors (SSRIs) and serotonin norepinephrine reuptake inhibitors (SNRIs) (Craske and Stein, 2016) is recommended. Therefore, careful considerations should be required for the continue usage of sulpiride for PUD and GERD.

Sulpiride obtained indications from Taiwan FDA to treat schizophrenia (dosage 300–600 mg/day, maximal dosage 1,200 mg/day), depression (dosage 150–300 mg/day, maximal dosage 600 mg/day), and gastric ulcer (dosage 150 mg/day) (Huang et al., 2019). Our study showed that an average annual sulpiride cumulative dosage of >1,103 mg granted the greatest risk of parkinsonism. Sulpiride used for >9 days or ≥6.75 cDDD is a cut-off point for predicting parkinsonism in the future. At the same time, the aHRs changed from 2.50 (95%CI = 1.51–4.13) to 3.20 (95%CI = 2.13–4.81) in cDDD from <6.75 to ≥6.75. It is easy to exceed the risk dosage, so our findings suggested that physicians should prescribe sulpiride in a short term and low dose manner to treat the PUD and GERD patients.

This study has certain limitations that should be considered while interpreting the results. Firstly, NHIRD does not contain the detailed information regarding diet, alcohol consumption, smoking habits, socioeconomic status, living environment, inactivity, or family history, despite the aforementioned factors being the potential risk factors for parkinsonism. Changes in these factors may affect the results. Secondly, although the secondary database research lacks important clinical information such as history, physical evaluation, and clinical course, some of the patients may have been wrongly classified. To eliminate this limitation, we excluded PUD or GERD subjects before sulpiride treatment with a history of PD, parkinsonism, stroke, dementia, head injury, and hydrocephalus. After sulpiride treatment for at least 14 days, we included subjects with PD and parkinsonism. Other limitations in our study that worth to be discussed may include: 1) Depression is recognized to occur as a first sign of parkinsonism, sometimes long before even detectable motor symptoms occur or being diagnosed (Lian et al., 2019). 2) In addition, anxiety can be an accompanying symptom of depression (Koutsimani et al., 2019). 3) There are still large number of patients with depression and anxiety, and their use of antidepressants like SSRIs and SNRIs may influence our conclusion, although our study excluded the individuals with antipsychotics treatment. Finally, all data in the NHIRD are anonymous. Therefore, relevant clinical variables such as body mass index, neuroimaging results, and serum laboratory data were unavailable for the study subjects. However, data related to sulpiride and parkinsonism diagnosis were highly reliable.

## Conclusion

In conclusion, sulpiride may be frequently prescribed and apparently effective for PUD and GERD. However, it is not included in clinical practice guidelines currently. SIP is associated with older age, and comorbidities of hypertension, depression, or anxiety. Parkinsonism could be induced, even exposing in a low-dose or a short duration. Physicians should be aware of the neurogenic adverse effects.

## Data Availability Statement

The dataset used in this study is held by the Taiwan Ministry of Health and Welfare (MOHW). MOHW must approve the application to access this dataset. Any researcher interested in accessing this dataset can submit an application form to MOHW requesting access. Please contact the staff of MOHW (Email: stcarolwu@mohw.gov.tw) for further assistance. The address of Taiwan Ministry of Health and Welfare is No.488, Sec. 6, Zhongxiao E. Rd., Nangang Dist., Taipei City 115, Taiwan (R.O.C.). Phone: +886-2-8590-6848. All relevant data are detailed in the manuscript.

## Ethics Statement

The studies involving human participants were reviewed and approved by The Research Ethics Committee of China Medical University and Hospital in Taiwan. Written informed consent for participation was not required for this study in accordance with the national legislation and the institutional requirements.

## Author Contributions

C-YW and W-MK proposed the research idea, wrote the results and discussion, and contributed to the literature review. M-CL performed the analysis. Y-HY and CYH supported the literature review and helped revise the manuscript. Y-HY and CYH provided clinical suggestions. I-ST supported data analysis and prepared the manuscript for submission. All authors read and approved the final manuscript.

## Conflict of Interest

The authors declare that the research was conducted in the absence of any commercial or financial relationships that could be construed as a potential conflict of interest.
